# Evaluation of urinary cysteinyl leukotrienes as biomarkers of severity and putative therapeutic targets in COVID-19 patients

**DOI:** 10.1007/s00011-022-01682-z

**Published:** 2023-01-08

**Authors:** Marta Reina-Couto, Mariana Roboredo-Madeira, Patrícia Pereira-Terra, Carolina Silva-Pereira, Sandra Martins, Luísa Teixeira-Santos, Dora Pinho, Andreia Dias, Gonçalo Cordeiro, Cláudia Camila Dias, António Sarmento, Margarida Tavares, João T. Guimarães, Roberto Roncon-Albuquerque, José-Artur Paiva, António Albino-Teixeira, Teresa Sousa

**Affiliations:** 1 Departamento de Biomedicina-Unidade de Farmacologia e Terapêutica, Faculdade de Medicina da Universidade do Porto (FMUP), Porto, Portugal; 2 Centro de Investigação Farmacológica e Inovação Medicamentosa da Universidade do Porto (MEDInUP), Porto, Portugal; 3 Serviço de Medicina Intensiva, Centro Hospitalar Universitário de São João (CHUSJ), Porto, Portugal; 4 Serviço de Farmacologia Clínica, CHUSJ, Porto, Portugal; 5 Serviço de Patologia Clínica, CHUSJ, Porto, Portugal; 6 Departamento de Medicina da Comunidade, Informação e Decisão em Saúde, FMUP, Porto, Portugal; 7 CINTESIS-Centro de Investigação em Tecnologias e Serviços de Saúde, Porto, Portugal; 8 Serviço de Doenças Infecciosas, CHUSJ, Porto, Portugal; 9 Departamento de Medicina, FMUP, Porto, Portugal; 10 EPIUnit, Instituto de Saúde Pública da Universidade do Porto, Porto, Portugal; 11 Departamento de Biomedicina-Unidade de Bioquímica, FMUP, Porto, Portugal; 12 Departamento de Cirurgia e Fisiologia, FMUP, Porto, Portugal

**Keywords:** Urinary cysteinyl leukotrienes, COVID-19, Disease severity, Veno-venous extracorporeal membrane oxygenation (VV-ECMO), Outcomes, Comorbidities

## Abstract

**Background:**

Cysteinyl leukotrienes (CysLT) are potent inflammation-promoting mediators, but remain scarcely explored in COVID-19. We evaluated urinary CysLT (U-CysLT) relationship with disease severity and their usefulness for prognostication in hospitalized COVID-19 patients. The impact on U-CysLT of veno-venous extracorporeal membrane oxygenation (VV-ECMO) and of comorbidities such as hypertension and obesity was also assessed.

**Methods:**

Blood and spot urine were collected in “severe” (*n* = 26), “critically ill” (*n* = 17) and “critically ill on VV-ECMO” (*n* = 17) patients with COVID-19 at days 1–2 (admission), 3–4, 5–8 and weekly thereafter, and in controls (*n* = 23) at a single time point. U-CysLT were measured by ELISA. Routine markers, prognostic scores and outcomes were also evaluated.

**Results:**

U-CysLT did not differ between groups at admission, but significantly increased along hospitalization only in critical groups, being markedly higher in VV-ECMO patients, especially in hypertensives. U-CysLT values during the first week were positively associated with ICU and total hospital length of stay in critical groups and showed acceptable area under curve (AUC) for prediction of 30-day mortality (AUC: 0.734, *p* = 0.001) among all patients.

**Conclusions:**

U-CysLT increase during hospitalization in critical COVID-19 patients, especially in hypertensives on VV-ECMO. U-CysLT association with severe outcomes suggests their usefulness for prognostication and as therapeutic targets.

**Supplementary Information:**

The online version contains supplementary material available at 10.1007/s00011-022-01682-z.

## Introduction

SARS-CoV-2 is a novel single-RNA encapsulated virus that is responsible for the recent pandemic disease known as COVID-19 [1, 2]. Patients with COVID-19 exhibit excessive inflammatory activity. This inflammatory response, frequently termed as “cytokine storm”, may worsen the progression of the infection through acute alveolar damage [3] and endothelial damage of the pulmonary vessels, with associated disseminated thrombosis, complement activation and microangiopathy [4, 5], leading to acute respiratory distress syndrome (ARDS), multiorgan damage and, eventually, death [6].

Although cytokines have been the most studied inflammatory mediators in COVID-19 pathophysiology, other mediators such as leukotrienes (LT) and cysteinyl leukotrienes (CysLT) have also potent inflammation-promoting actions that may be of special relevance in this clinical context [7]. CysLT are known for their role in inflammation in diseases such as asthma and allergic rhinitis [8, 9], being powerful bronchoconstrictors in humans. Indeed, they exert several actions relevant for respiratory pathology, being able to stimulate mucous secretion and bronchial hyperresponsiveness, increase microvasculature permeability and stimulate eosinophil infiltration [10–12]. In addition, our group recently showed that urinary CysLT were positively correlated with endothelial activation, inflammation, oxidative stress, and hepatic dysfunction markers in septic shock patients [13]. CysLT, namely leukotriene C_4_ (LTC_4_), leukotriene D_4_ (LTD_4_) and leukotriene E_4_ (LTE_4_), are arachidonic acid-derived eicosanoids synthesized by the 5-lipoxygenase (5-LOX)/LTC_4_ synthase pathway in immunocompetent cells (e.g., mast cells, neutrophils, eosinophils, basophils and monocytes) [14, 15]. In the circulation, LTC_4_ and LTD_4_ are rapidly metabolized to LTE_4_, which also has a short half-life [16, 17]. LTE_4_ is excreted in urine and changes in its urinary excretion are considered to be evidence of short-term changes in the formation rate of LTC_4_ [18]. The G protein-coupled CysLT_1_ and CysLT_2_ receptors, expressed in the outer cell membrane of immune and inflammatory cells, endothelial cells and platelets, mediate most CysLT effects relevant for immune responses, namely macrophage activation, inflammatory cytokine release and activation of the transcription factor nuclear factor kappa B (NF-κB) that regulates various genes related to inflammation, contributing overall to a hyperinflammatory and immune activation state [9, 19–21]. Due to the similarities between COVID-19-associated and non-associated respiratory pathology, there is a likely association between CysLT and the pulmonary pathology of COVID-19 [22]. However, they remain scarcely explored in this disease.

For all these reasons, we aimed to evaluate the relationship between CysLT profile and COVID-19 severity, focusing on their role as biomarkers for risk stratification and prognostication, as well as their plausibility for eventual therapeutic targeting. Importantly, we also assessed the impact of comorbidities associated with higher risk of critical disease and death, such as arterial hypertension and obesity [23–27], in severe and critical COVID-19 patients, as well as the effect of veno-venous extracorporeal membrane oxygenation (VV-ECMO) on CysLT, which has never been reported so far.

## Materials and methods

### Study design and population

The present study is part of a larger research project (RESEARCH 4 COVID-19 grant, project 519-reference number 613690173, “Unresolved inflammation and endotheliitis in severe COVID-19 patients: identification of risk stratification biomarkers and therapeutic targets”, FCT—Fundação para a Ciência e a Tecnologia) involving patients from the ward of the Service of Infectious Diseases and from the Intensive Care Unit (ICU) of the Service of Intensive Care Medicine and the Service of Infectious Diseases of a tertiary hospital (Centro Hospitalar Universitário de São João, CHUSJ). From September 2020 to February 2021, we consecutively recruited patients with a laboratory-confirmed diagnosis of SARS-CoV-2 infection, defined by a positive result on an RT-PCR assay of a specimen collected on a nasopharyngeal swab, who were hospitalized in the context of hypoxemic respiratory failure and symptomatic for > 1 day. Patients were excluded if they were under 18 years of age, were pregnant or lactating or had a history of vasculitis or connective tissue disease. Sixty-one patients (*n* = 61) were enrolled in this single-center cohort study, with the majority of them being recruited within 72 h of a positive RT-PCR result. Admission to the ward or ICU and the time for intubation and mechanical ventilation or VV-ECMO was based on clinical judgment according to *leges artis*. Patients were divided into two groups according to COVID-19 disease severity [28]: patients with severe COVID-19 admitted to the ward (*n* = 27) and patients with critical COVID-19 admitted to the ICU (*n* = 34). Severe COVID-19 was characterized by the presence of oxygen saturation < 90% on room air, signs of pneumonia or signs of severe respiratory distress, whereas critical disease was defined as patients presenting criteria for Acute Respiratory Distress Syndrome (ARDS), sepsis, septic shock, or other conditions that require life-sustaining therapies, according to the World Health Organization’s latest guidelines [28]. For analysis purposes, we only included the patients in whom we were able to collect both blood and spot urine samples at admission. In the severe COVID-19 group, we excluded one patient who lacked a urine sample at admission. Therefore, we had a final number of 26 severe COVID-19 patients and 34 patients with critical COVID-19. The group of patients with critical COVID-19 was further subdivided into two groups depending on whether or not they received VV-ECMO support: group of critically ill COVID-19 patients on VV-ECMO (critical COVID-19 on VV-ECMO, *n* = 17) and group of critically ill COVID-19 patients without VV-ECMO support (critical COVID-19, *n* = 17).

Due to the prospective nature of our sampling, we were able to capture a heterogeneous population of ward patients and ICU patients, some recruited before or during admission to ICU. Controls (*n* = 23) were recruited among healthy blood donor volunteers from the Service of Immunohemotherapy of CHUSJ before the COVID-19 pandemic. All eligible patients provided written informed consent to participate in the study. For ICU patients unable to give consent, this was solicited from their next of kin. These patients provided informed consent retrospectively, whenever possible. Blood donor volunteers provided oral informed consent. The study was conducted in accordance with the Guidelines for Good Clinical Practice and the 1975 Declaration of Helsinki after approval by the CHUSJ Health Ethics Committee [CES 75-16, with project amended specifically for inclusion of subjects with COVID-19, within the scope of a RESEARCH 4 COVID-19 grant from FCT (special support for rapid implementation projects for innovative response solutions to COVID-19 pandemic)].

### Clinical data and sample collection

Patients were followed during their stay in the ward or ICU by the medical team of the project.

Throughout their hospitalization, regular physical examination of the patients was performed to assess major vital and clinical signs. For each patient, the medical team assessed the data regarding clinical and relevant demographic parameters, which were further anonymously coded to the project database, along with routine laboratory data, guaranteeing confidentiality. Illness severity was assessed by the Acute Physiology and Chronic Health Evaluation II (APACHE II) and Simplified Acute Physiology Score II (SAPS II) scoring systems at ICU admission. ICU length of stay, total hospital length of stay and mortality at 30 days were also evaluated. The group of patients with critical COVID-19 on VV-ECMO included some patients who were previously hospitalized in the ICU of other hospitals before admission to the ICU of CHUSJ and that period was counted for the calculation of ICU length of stay. In addition, all patient groups included a few patients who were further transferred from CHUSJ to other hospitals and all consecutive period of hospitalization was counted for calculation of total hospital length of stay.

For all patients, blood and spot urine samples were collected at different time points throughout their hospital stay at CHUSJ, whenever possible: up to 48 h (days 1–2; admission), on days 3–4, on days 5–8 after admission and weekly thereafter until hospital discharge or after a negative result in RT-PCR COVID-19 test. All collections of critical COVID-19 patients on VV-ECMO were started after VV-ECMO initiation. Samples (blood and spot urine) from controls were collected at a single time point. All samples were processed within 1–2 h of collection and stored at − 80ºC until assayed.

### Quantification of routine markers

Most routine laboratory analyses were performed at the Service of Clinical Pathology of CHUSJ. An Olympus Beckman Coulter® AU5400 automated clinical chemistry analyser (Olympus, Hamburg, Germany) was used for the quantification of serum CRP (S-CRP) by an immunoturbidimetric assay, serum aspartate aminotransferase (AST) and alanine aminotransferase (ALT) by kinetic photometric assays, gamma glutamyltransferase (G-GT) by a kinetic colorimetric method, serum alkaline phosphatase (ALP), total and direct bilirubin and albumin by colorimetric assays, serum LDH (S-LDH) by a spectrophotometric assay and plasma and urinary creatinine by the colorimetric Jaffe method. Differential leukocyte count (leukocytes, neutrophils, eosinophils, monocytes and lymphocytes) was analyzed by flow-cytometry in an automated hematology analysis system (Sysmex 5000; Emilio de Azevedo Campos, Porto, Portugal), with subsequent calculation of neutrophil-to-lymphocyte ratio (NLR) and neutrophil-to-monocyte ratio (NMR).

Quantification of lactate, partial pressure of oxygen (PaO_2_) and partial pressure of carbon dioxide (PaCO_2_) was performed by arterial blood gas analysis. Fraction of inspired oxygen (FiO_2_) was obtained from oxygen administration device and oxygen dose information in the medical records and the PaO_2_/FiO_2_ ratio was calculated.

### Quantification of urinary cysteinyl leukotrienes

CysLT were measured in unextracted spot urine samples (U-CysLT) by a competitive enzyme-linked immunosorbent assay (ELISA) using a commercial kit (Cysteinyl Leukotriene ELISA kit, Cayman Chemical Company, Ann Arbor, MI, USA). The values of U-CysLT measured by this kit were previously shown to be well correlated with the values obtained by ultra-performance liquid chromatography triple quadrupole mass spectrometry (UPLC-MS/MS) [29]. U-CysLT concentrations were further corrected for urinary creatinine values.

### Quantification of proinflammatory cytokines

Serum proinflammatory cytokines (serum tumor necrosis factor alpha, S-TNF-α; serum interleukin 1 beta, S-IL-1β; serum IL-6, S-IL-6) were evaluated by multiplex immunoassays using a Luminex 200TM xMAP™ analyser (Luminex Corporation, Austin, TX, USA), according to the protocol of Human High Sensitivity T Cell Magnetic Bead Panel (Milliplex^®^ Map kit, Millipore Corporation, Billerica, MA, USA). Raw data analysis (mean fluorescence intensity) was performed using a standard five parameter logistic (5-PL) curve fit created by the Luminex xPONENT^®^ Software (version 3.1).

### Statistical analysis

Results are expressed as mean ± standard error of the mean (SEM) or as median (25th percentile; 75th percentile) for data with normal or non-normal distribution, respectively, or as percentage. Results are graphically represented as Box-and-Whiskers plots. Estimated glomerular filtration rate (eGFR) was calculated using the Chronic Kidney Disease Epidemiology Collaboration (CKD-EPI) equation [30] Statistical analysis was conducted using the GraphPad Prism 9 software (GraphPad Software, La Jolla, California, USA). Results were analyzed by unpaired Student’s *t* test or Mann–Whitney *U* test, for comparisons between two groups, or by one-way ANOVA followed by a Tukey’s multiple comparison test or a Kruskal–Wallis test followed by a Dunn’s post hoc test, for comparison between three groups, where appropriate. Categorical variables were analyzed by the Chi-Square test. Biomarkers evolution throughout the hospitalization was analyzed by Wilcoxon matched pairs signed rank test. Due to scarcity of samples at later time points, statistical analysis was only possible for results obtained until week 5 of hospitalization. Spearman’s correlation analysis was used to estimate correlations between sets of nonparametric data among all critically ill patients (critical COVID-19 and critical COVID-19 on VV-ECMO patients) throughout the first week of hospitalization (admission; days 3–4; days 5–8).

Repeated measures multivariate analyses were conducted to determine the relationship between U-CysLT (as dependent variable) and the COVID-19 patient group or patient comorbidities, adjusted for some confounders such as age and gender, among all COVID-patients during the first week of hospitalization, or between U-CysLT (as dependent variable) and hypertension, previous RAAS inhibitor treatment, eGFR, ICU length of stay, total hospital length of stay and 30-day mortality, adjusted for age and gender, among all critically ill patients during the first week of hospitalization.

The ability of U-CysLT to discriminate 30-day mortality in all COVID-19 patients or only in critical COVID-19 patient groups was evaluated by plotting receiver operating characteristic (ROC) curves and computing the area under the curve (AUC). All *p* values of < 0.050 were considered significant.

To prevent possible bias in clinical evaluation, all the patients were examined by the same medical team included in the project. To assure comparability of biomarkers assessment, samples from controls, severe COVID-19, critical COVID-19 and critical COVID-19 on VV-ECMO groups were evenly distributed in each assay plate. There were missing values in some biomarkers due to insufficient volume to perform sample processing, dilution tests and assays. We had no permission to measure routine clinical biomarkers in controls (blood donor volunteers), except for creatinine, or to have access to their hospital laboratory reports or clinical records. The final number per group for the biomarkers/parameters evaluated at admission was as following: APACHE II, *n* = 17 vs *n* = 17 (Critical vs Critical on VV-ECMO); SAPS II, *n* = 17 vs *n* = 17 (Critical vs Critical on VV-ECMO); lactate, *n* = 20 vs *n* = 15 vs *n* = 16 (Severe vs Critical vs Critical on VV-ECMO); PaO_2_/FiO_2_ Ratio, *n* = 26 vs *n* = 17 vs *n* = 17 (Severe vs Critical vs Critical on VV-ECMO); PaCO_2_, *n* = 24 vs *n* = 17 vs *n* = 17 (Severe vs Critical vs Critical on VV-ECMO); AST, *n* = 25 vs *n* = 16 vs *n* = 13 (Severe vs Critical vs Critical on VV-ECMO); ALT, *n* = 25 vs *n* = 15 vs *n* = 13 (Severe vs Critical vs Critical on VV-ECMO); ALP, *n* = 24 vs *n* = 16 vs *n* = 13 (Severe vs Critical vs Critical on VV-ECMO); G-GT, *n* = 24 vs *n* = 16 vs *n* = 14 (Severe vs Critical vs Critical on VV-ECMO); total bilirubin, *n* = 24 vs *n* = 16 vs *n* = 15 (Severe vs Critical vs Critical on VV-ECMO); direct bilirubin, *n* = 17 vs *n* = 10 vs *n* = 10 (Severe vs Critical vs Critical on VV-ECMO); LDH, *n* = 24 vs *n* = 8 vs *n* = 5 (Severe vs Critical vs Critical on VV-ECMO); albumin, *n* = 19 vs *n* = 14 vs *n* = 15 (Severe vs Critical vs Critical on VV-ECMO); S-CRP, *n* = 26 vs *n* = 17 vs *n* = 17 (Severe vs Critical vs Critical on VV-ECMO); S-TNF-α, *n* = 23 vs *n* = 26 vs *n* = 17 vs *n* = 17 (Controls vs Severe vs Critical vs Critical on VV-ECMO); S-IL-1β, *n* = 23 vs *n* = 26 vs *n* = 17 vs *n* = 17 (Controls vs Severe vs Critical vs Critical on VV-ECMO); S-IL-6, *n* = 22 vs *n* = 26 vs *n* = 17 vs *n* = 17 (Controls vs Severe vs Critical vs Critical on VV-ECMO); leukocytes, *n* = 26 vs *n* = 17 vs *n* = 16 (Severe vs Critical vs Critical on VV-ECMO); neutrophils, *n* = 26 vs *n* = 17 vs *n* = 15 (Severe vs Critical vs Critical on VV-ECMO); eosinophils, *n* = 26 vs *n* = 17 vs *n* = 15 (Severe vs Critical vs Critical on VV-ECMO); monocytes, *n* = 26 vs *n* = 17 vs *n* = 15 (Severe vs Critical vs Critical on VV-ECMO); lymphocytes, *n* = 26 vs *n* = 17 vs *n* = 15 (Severe vs Critical vs Critical on VV-ECMO); NLR (neutrophils/lymphocytes ratio), *n* = 26 vs *n* = 17 vs *n* = 15 (Severe vs Critical vs Critical on VV-ECMO); NMR (neutrophils/monocytes, *n* = 26 vs *n* = 17 vs *n* = 15 (Severe vs Critical vs Critical on VV-ECMO). In addition, the number of patients in each group decreased throughout hospitalization due to death, withdrawal of consent, hospital discharge or a negative RT-PCR COVID-19 test (Supplementary Figs. 1–3). Moreover, there were some patients in whom it was not possible to collect blood and/or urine samples at all time points throughout hospitalization due to medical/nurse team logistics or due to the lack of a sufficient urine excretion by the patients (in the case of urine collection) (Supplementary Figs. 1–3). To avoid biasing the results, no imputation for missing values was used.

Sample size was defined according to the primary objectives of our FCT funded RESEARCH 4 COVID-19 project that consisted in characterizing resolution of inflammation and endotheliitis. Based on preliminary evaluations of specialized proresolving mediators and endocan in healthy controls, patients with severe disease and critically ill patients, using power analysis, we calculated a sample size of 21 subjects per group to obtain an 80% power, at a 5% significance level (effect size-to-standard deviation ratio ca. 0.9). Since there was an elevated number of critically ill patients on VV-ECMO and a high heterogeneity of values between critically ill patients without VV-ECMO support vs those on VV-ECMO, we further divided the group of patients with critical COVID-19 into two groups: critically ill (without VV- ECMO) and critically ill on VV-ECMO. Despite this change, we had a total sample size of 83 subjects (i.e., about 4 times the 21 initially estimated), albeit with only 17 patients per group in the 2 critically ill groups. Reporting of the study conforms to STROBE statement along with references to STROBE and the broader EQUATOR guidelines [31].

## Results

### Population demographic, clinical and biochemical characterization

Demographic, clinical and biochemical characteristics of the subjects included in the study are presented in Table 1. In this study, 23 healthy controls and 60 patients, 26 with severe COVID-19, 17 with critical COVID-19 and 17 with critical COVID-19 on VV-ECMO were assessed. Patients with severe COVID-19 were older than controls (*p* < 0.010), while critically ill COVID-19 on VV-ECMO patients were significantly younger than severe and critically ill COVID-19 patients (*p* < 0.001 and *p* < 0.050, respectively). There was a male predominance in all groups, but there were no significant gender differences between groups. Arterial hypertension was the most prevalent comorbidity in severe and critically ill COVID-19 patients, while obesity was the most prevalent in critical COVID-19 on VV-ECMO patients, followed by arterial hypertension, although no significant differences were found between patient groups. We did not find differences in APACHE II and SAPS II scores between the groups of critical patients. Lactate concentration at admission also did not differ between the patient groups.

**Table 1:** Demographic, clinical and biochemical characteristics at admission and follow-up parameters of the study population

Demographic, clinical and biochemical parameters	Controls (*n* = 23)	Severe COVID-19 (*n* = 26)	Critical COVID-19 (*n* = 17)	Critical COVID-19 on VV-ECMO (*n* = 17)	*p* value
**Age (years)**	**57 (53; 63)**	71 (62; 80)**	**67 (55; 72)**	**55** **(40; 59)**^**###,†**^	**< 0.001**
Gender: men, *n* (%)	15 (65)	16 (62)	11 (65)	11 (65)	0.993
Gender: women, *n* (%)	8 (35)	10 (38)	6 (35)	6 (35)	0.993
Comorbidities, *n* (%)					
Diabetes	n.d.	10 (38)	6 (35)	4 (24)	0.585
Obesity	n.d.	7 (27)	8 (47)	10 (59)	0.101
Arterial hypertension	n.d.	18 (69)	13 (76)	8 (47)	0.166
Heart failure	n.d.	6 (23)	3 (18)	1 (6)	0.332
Respiratory disease	n.d.	8 (31)	4 (24)	2 (12)	0.354
Renal disease	n.d.	6 (23)	4 (24)	0 (0)	0.093
Malignancy	n.d.	2 (8)	0 (0)	0 (0)	0.259
AIDS	n.d.	1 (4)	0 (0)	0 (0)	0.514
APACHE II score	n/a	n/a	17 ± 2	19 ± 2	0.423
SAPS II score	n/a	n/a	42 ± 4	40 ± 4	0.666
Lactate (mmol/L)	n/a	1.1 (1.0; 1.6)	1.5 (1.1; 1.8)	1.5 (1.2; 1.7)	0.262
Previous therapeutics, *n* (%)					
**RAAS inhibitors prior to admission**	n/a	**15 (58)**	**12 (71)**	**4 (24)**	**0.017**
**Hypertensive patients on RAAS prior to admission**	n/a	**14 (54)**	**11 (65)**	**4 (24)**	**0.042**
Therapeutics at admission, *n* (%)					
Dexamethasone	n/a	20 (77)	16 (94)	16 (94)	0.152
Remdesivir	n/a	1 (4)	0 (0)	2 (12)	0.272
Antibiotics	n/a	5 (19)	7 (41)	9 (53)	0.063
Respiratory parameters					
**PaO**_**2**_/**FiO**_**2**_ **ratio**	n/a	**260 (225; 288)**	**92 (68; 137)**^**###**^	**100 (76; 119)**^**###**^	**< 0.001**
**PaCO**_**2**_ **(mmHg)**	n/a	**32 ± 1**	**37 ± 1**	**48 ± 2**^**###**^	**< 0.001**
Hepatic biomarkers					
AST (U/L)	n/a	45 (31; 70)	39 (30; 52)	49 (31; 66)	0.629
ALT (U/L)	n/a	34 (28; 54)	27 (20; 59)	44 (23; 63)	0.487
ALP (U/L)	n/a	77 (61; 116)	68 (54; 83)	80 (53; 117)	0.314
**G-GT (U/L)**	n/a	**63 (28; 149)**	**56 (32; 132)**	**132 (107; 187)**^**#,†**^	**0.017**
Total bilirubin (mg/dL)	n/a	0.7 (0.5; 0.9)	0.6 (0.5; 0.9)	0.7 (0.5; 1.1)	0.692
**Direct bilirubin (mg/dL)**	n/a	**0.2 (0.1; 0.3)**	**0.2 (0.1; 0.3)**	**0.4 (0.2; 0.7)**	**0.049**
**Albumin (g/L)**	n/a	**33 (29; 36)**	**30 (29; 32)**	**25 (22; 27)**^**###,†**^	**< 0.001**
LDH (U/L)	n/a	355 (279; 457)	441 (333; 570)	558 (395; 573)	0.149
Inflammatory status					
**S-CRP (mg/L)**	n.d.	**100 (49; 174)**	**116 (78; 190)**	**163 (116; 245)**^#^	**0.013**
**S-TNF-α (pg/mL)**	**11 (7; 15)**	20 (13; 32)**	26 (19; 37)***	22 (14; 30)**	**< 0.001**
** S-IL-1β (pg/mL)**	**0.3 (0.0; 0.7)**	**0.8 (0.3; 1.6)**	1.7 (1.2; 2.5)**	1.3 (0.8; 2.7)**	**< 0.001**
**S-IL-6 (pg/mL)**	**0 (0; 3)**	9 (5; 19)***	15 (6; 52)***	27 (4; 143)***	**< 0.001**
**Leukocytes (× 10**^**9**^**/L**)	n.d.	**6 (5; 10)**	**9 (6; 12)**^**#**^	**12 (9; 13)**^**###**^	**< 0.001**
**Neutrophils (× 10**^**9**^**/L)**	n.d.	**4 (3; 8)**	**9 (6; 12)**^**##**^	**10 (8; 12)**^**###**^	**< 0.001**
**Eosinophils (× 10**^**9**^**/L)**	n.d.	**0.00 (0.00; 0.01)**	**0.00 (0.00; 0.00)**	**0.02 (0.00; 0.11)**^**†††**^	**< 0.001**
Monocytes (× 10^9^/L)	n.d.	0.4 ± 0.0	0.4 ± 0.1	0.5 ± 0.1	0.228
Lymphocytes (× 10^9^/L)	n.d.	0.9 ± 0.1	0.8 ± 0.1	1.1 ± 0.1	0.216
**NLR**	n.d.	**5 (3; 8)**	**12 (8; 17)**^**###**^	**10 (7; 13)**^**#**^	**< 0.001**
**NMR**	n.d.	**11 (9; 20)**	**20 (15; 42)**^**#**^	**16 (14; 36)**	**0.008**
Follow-up					
Type of oxygen support during hospitalization, *n* (%)					
**Mechanical ventilation**	n/a	**2 (8)**	**11 (65)**	**17 (100)**	**< 0.001**
**NIV**	n/a	**5 (19)**	**11 (65)**	**14 (82)**	**< 0.001**
** High-flow cannula**	n/a	**9 (35)**	**13 (76)**	**9 (53)**	**0.027**
Supplementary oxygen	n/a	25 (96)	13 (76)	16 (94)	0.088
**ICU length of stay (days)**	n/a	**0 (0; 0)**	**16 (7; 33)**^**###**^	**34 (16; 74)**^**###**^	**< 0.001**
**Total hospital length of stay (days)**	n/a	**7 (5; 15)**	**22 (11; 57)**^**##**^	**43 (25; 116)**^**###**^	**< 0.001**
Mortality within 30 days, *n* (%)	n/a	3 (12)	4 (24)	1 (6)	0.298

Results are expressed as number (%), mean ± SEM or as median (25th percentile; 75th percentile) for data with normal or non-normal distribution, respectively

*AIDS* Acquired Immunodeficiency Syndrome, *ALP* alkaline phosphatase, *ALT* alanine transaminase, *APACHE II* acute physiology and chronic health evaluation II, *AST* aspartate aminotransferase, *FiO*_*2*_ fraction of inspired oxygen, *G-GT* gamma-glutamyl transferase, *ICU* intensive care unit, *LDH* lactate dehydrogenase, *n.d.* not determined, *n/a* not applicable, *NIV* non-invasive ventilation, *NIV* non-invasive ventilation, *NLR* neutrophil–lymphocyte ratio, *NMR* neutrophil–monocyte ratio, *PaCO*_*2*_ partial pressure of arterial carbon dioxide, *PaO*_*2*_ partial pressure of arterial oxygen, *SAPS II* simplified acute physiology score II, *S-CRP* serum C-reactive protein; VV-ECMO, veno-venous extracorporeal membrane oxygenation

**p < 0.01 vs Controls; ***p < 0.001 vs Controls; ^#^*p* < 0.05 vs Severe; ^##^*p* < 0.01 vs Severe; ^###^*p* < 0.001 vs Severe; ^†^*p* < 0.05 vs Critical; ^†††^*p* < 0.001 vs Critical. All parameters with statistically significant differences between groups are highlighted in bold

Regarding respiratory function, PaCO_2_ was significantly higher only in patients with COVID-19 on VV-ECMO (*p* < 0.001 vs severe COVID-19) and the PaO_2_/FiO_2_ ratio was significantly lower in both groups of critically ill patients compared to patients with severe COVID-19 (*p* < 0.001). Accordingly, and by definition, there was a higher need of mechanical ventilation, non-invasive ventilation and high-flow cannula oxygen in all critical COVID-19 patients (with or without VV-ECMO) when compared with severe COVID-19 patients (*p* < 0.001, *p* < 0.001 and *p* = 0.027 respectively).

There was a significant difference between groups regarding the number of patients who were previously treated with RAAS inhibitors (*p* = 0.017), with the critical COVID-19 on VV-ECMO group including a lower proportion (24%) of patients receiving a RAAS inhibitor prior to admission, compared to the severe and critical groups (58% and 71%, respectively). In both groups of critically ill patients, the RAAS inhibitor treatment was interrupted during hospitalization. Regarding the therapeutics initiated at admission, almost all patients were treated with dexamethasone and very few were treated with remdesivir with no differences between groups. There was a higher proportion of critically ill COVID-19 patients (with or without VV-ECMO) receiving antibiotics compared to severe patients, although this difference was not statistically significant (*p* = 0.063).

All COVID-19 patient groups exhibited significantly higher concentrations of inflammatory cytokines such as S-TNF-α, S-IL-1β, and S-IL-6 compared to controls. S-CRP concentration was higher in patients with critical COVID-19 on VV-ECMO compared to severe COVID-19 patients (*p* < 0.013). Patients with critical COVID-19 or with critical COVID-19 on VV-ECMO also showed significantly higher leukocyte count when compared to severe COVID-19 patients (*p* < 0.05 and *p* < 0.001, respectively), as well as higher neutrophil count (*p* < 0.010 and *p* < 0.001 vs severe COVID-19, respectively). Critical COVID-19 on VV-ECMO patients had higher eosinophil count when compared to critical COVID-19 group (*p* < 0.001). No differences were observed for monocyte and lymphocyte counts, but NLR was significantly higher in critical COVID-19 group and in patients with critical COVID-19 on VV-ECMO when compared to severe COVID-19 patients (*p* < 0.001 and *p* < 0.050, respectively) and NMR was significantly higher in patients with critical COVID-19 compared to severe COVID-19 patients (*p* < 0.05).

Patients with critical COVID-19 on VV-ECMO exhibited higher values of hepatic cholestatic parameters, namely G-GT (*p* < 0.050 vs severe COVID-19 and critical COVID-19) and direct bilirubin (*p* = 0.049). Critically ill patients on VV-ECMO also had significantly lower values of albumin when compared to both severe and critical COVID-19 groups (*p* < 0.001 and *p* < 0.050, respectively). No other significant differences were found between the three groups concerning any other hepatic parameters.

Both groups of critically ill patients had a longer length of stay in the ICU than severe patients (*p* < 0.001), taking into account that five severe COVID-19 patients needed a temporary upgrade of care to ICU in the first week of hospitalization [median ICU length of stay (25th percentile; 75th percentile): 11 (3; 36) days]. At ICU admission, those five patients had mean (± S.E.M.) APACHE II and SAPS II scores of 11 ± 2 and 28 ± 10, respectively. Moreover, the group of critical COVID-19 on VV-ECMO had a longer length of stay in ICU than the critical COVID-19 group, although this difference was not statistically significant. Both groups of critically ill patients had a longer total hospital length of stay when compared to severe COVID-19 patients (*p* < 0.001 and *p* < 0.010, respectively). No significant differences were observed in mortality within 30 days between COVID-19 patient groups in this population sample.

### U-CysLT in all groups at admission

**Fig. 1:**
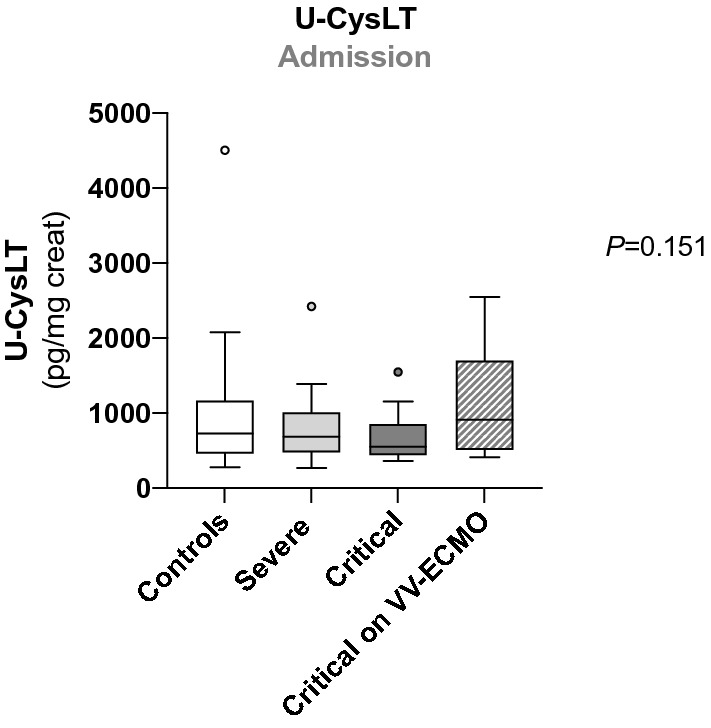
U-CysLT in all groups at admission. Results are presented in Box-and-Whiskers plot, controls (*n* = 23), severe (*n* = 26), critical (*n* = 17), critical on VV-ECMO (*n* = 17). *U-CysLT* urinary cysteinyl leukotrienes, *VV-ECMO* veno-venous extracorporeal membrane oxygenation

There were no significant differences in admission U-CysLT values between groups (*p* = 0.151) (Fig. 1).

### Comparison of U-CysLT between COVID-19 patient groups during the first week of hospitalization

**Fig. 2:**
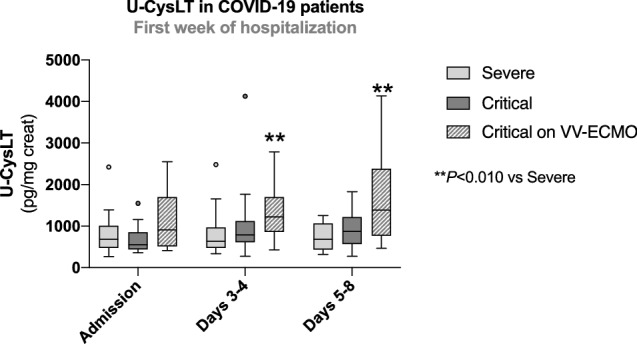
Comparison of U-CysLT between COVID-19 patient groups during the first week of hospitalization. Results are presented in Box-and-Whiskers plot, severe: admission (*n* = 26), days 3–4 (*n* = 20), days 5–8 (*n* = 15); critical: admission (*n* = 17), days 3–4 (*n* = 16), days 5–8 (*n* = 13); critical on VV-ECMO: admission (*n* = 17), days 3–4 (*n* = 17), days 5–8 (*n* = 17). *U-CysLT* urinary cysteinyl leukotrienes, *VV-ECMO* veno-venous extracorporeal membrane oxygenation

Patients with critical COVID-19 on VV-ECMO showed markedly higher U-CysLT values than severe COVID-19 patients at days 3–4 (*p* < 0.010) and at days 5–8 (*p* < 0.010) (Fig. 2).

### U-CysLT profiles throughout hospitalization

**Fig. 3:**
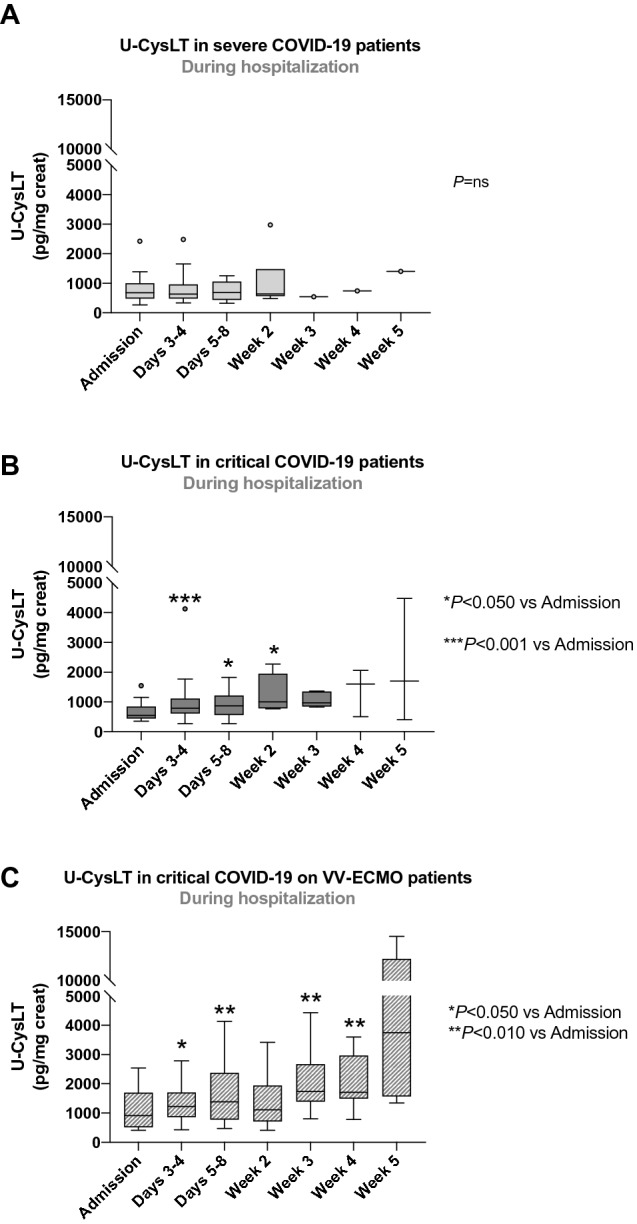
U-CysLT profiles throughout hospitalization in patients with severe COVID-19 (**A**), critical COVID-19 (**B**) and critical COVID-19 on VV-ECMO (**C**). Results are presented in Box-and-Whiskers plot, severe: admission (*n* = 26), days 3–4 (*n* = 20), days 5–8 (*n* = 15), week 2 (*n* = 7), week 3 (*n* = 1), week 4 (*n* = 1), week 5 (*n* = 1); critical: admission (*n* = 17), days 3–4 (*n* = 16), days 5–8 (*n* = 13), week 2 (*n* = 7), week 3 (*n* = 5), week 4 (*n* = 3), week 5 (*n* = 3); critical on VV-ECMO: admission (*n* = 17), days 3–4 (*n* = 17), days 5–8 (*n* = 17), week 2 (*n* = 13), week 3 (*n* = 10), week 4 (*n* = 9), week 5 (*n* = 4). *U-CysLT* urinary cysteinyl leukotrienes, *VV-ECMO* veno-venous extracorporeal membrane oxygenation

In severe COVID-19 patients, U-CysLT values remained unchanged during hospitalization (Fig. 3A). On the other hand, both patients with critical COVID-19 and critical COVID-19 on VV-ECMO presented a rising pattern of U-CysLT. In the critical COVID-19 group, these values were significantly higher from days 3–4 until week 2 compared to admission (Fig. 3B), while in critical COVID-19 on VV-ECMO group, U-CysLT values were higher from days 3–4 until week 4 when compared to values on admission (Fig. 3C).

### Impact of arterial hypertension and obesity on U-CysLT during the first week of hospitalization

In the critical COVID-19 on VV-ECMO group, U-CysLT values were higher in hypertensive patients compared to normotensive ones, but these differences only achieved statistical significance at days 5–8 (*p* = 0.027) (Table2). Regarding severe and critical COVID-19 patients, no significant differences on U-CysLT values were found between hypertensive and normotensive patients at any time point (Table 2).

**Table 2:** Impact of arterial hypertension and obesity on urinary cysteinyl leukotrienes at admission, days 3–4 and 5–8 in patients with severe COVID-19, critical COVID-19 and critical COVID-19 on VV-ECMO patients

Hypertension									
	Admission			Days 3–4			Days 5–8		
	Normotensive	Hypertensive	*p*	Normotensive	Hypertensive	*p*	Normotensive	Hypertensive	*p*
Severe COVID-19									
U-CysLT (pg/mg creatinine)	784 (542; 994)	637 (440; 1009)	0.531	501 (374; 1597)	663 (490; 872)	0.735	514 (354; 1091)	702 (492; 1065)	0.594
Critical COVID-19									
U-CysLT (pg/mg creatinine)	467 (390; 968)	581 (491; 851)	0.549	790 (400; 3293)	780 (611; 1123)	0.953	757 (271; 1243)	871 (602; 1193)	0.769
Critical COVID-19 on VV-ECMO									
U- CysLT (pg/mg creatinine)	520 (476; 1588)	1000 (755; 1820)	0.200	999 (668; 1508)	1502 (990; 2052)	0.167	**852 (528**; **1484)**	**2129 (1432**;** 2988)**	**0.027**

Obesity									
	Admission			Days 3–4			Days 5–8		
	Non-obese	Obese	*p*	Non-obese	Obese	*p*	Non-obese	Obese	*p*
Severe COVID-19									
U-CysLT (pg/mg creatinine)	740 (535; 1011)	487 (281; 686)	0.083	663 (482; 1269)	604 (443; 807)	0.485	718 (436; 1070)	628 (382; 969)	0.661
Critical COVID-19									
U-CysLT (pg/mg creatinine)	**637 (548**; **1077)**	**441(377**; **576)**	**0.021**	796 (742; 1367)	647(461; 919)	0.211	1142 (602; 1442)	750 (458; 952)	0.138
Critical COVID-19 on VV-ECMO									
U- CysLT (pg/mg creatinine)	1459 (493; 1765)	870 (568; 1279)	0.887	1223 (797; 2067)	1199 (900; 1694)	> 0.999	1327 (541; 1996)	1476 (1040; 2660)	0.669

*U-CysLT* urinary cysteinyl leukotrienes, *VV-ECMO* veno-venous extracorporeal membrane oxygenation. Results are expressed as median (25th percentile; 75th percentile). All values within each COVID-19 patient group (Severe COVID-19; Critical COVID-19; Critical COVID-19 on VV-ECMO) and within each time point (Admission; Days 3-4; Days 5-8) presenting statistically significant differences between subgroups (Normotensive vs Hypertensive; Non-obese vs Obese) are highlighted in bold

Obesity was not associated with higher U-CysLT. In fact, in patients with critical COVID-19, we observed significantly lower admission U-CysLT values in obese subjects compared to non-obese ones (*p* = 0.021) (Table 2). No significant differences between obese and non-obese subjects were observed at other time points in severe, critical or critical COVID-19 on VV-ECMO patients (Table 2).

### Correlation analyses in all critically ill patients during the first week of hospitalization

U-CysLT values were positively correlated with lactate at admission and were associated with a poorer respiratory function, evidenced by their positive correlation with PaCO_2_ at admission and inverse correlation with PaO_2_/FiO_2_ at days 3–4. U-CysLT were also associated with hepatic injury/dysfunction throughout the first week of hospitalization, presenting inverse correlations with albumin and positive correlations with AST and ALP. U-CysLT were also positively correlated with several inflammatory markers/parameters, namely S-CRP, S-TNF-α, S-IL-6, leukocyte count, neutrophil count, eosinophil count and NMR. Finally, we also observed positive correlations between U-CysLT during the first week of hospitalization and the hospital length of stay (both ICU and total length of stay) (Table 3).

**Table 3:** Correlations of urinary cysteinyl leukotrienes with clinical parameters and biomarkers in all critically ill patients during the first week of hospitalization

	Admission		Days 3–4		Days 5–8	
	*r* Spearman	*p*	*r* Spearman	*p*	*r* Spearman	*p*
APACHE II score	0.320	0.065	–	–	–	–
SAPS II score	0.267	0.126	–	–	–	–
Lactate (mmol/L)	**0.407**	**0.023**	− 0.109	0.581	− 0.311	0.121
PaO_2_/FiO_2_ ratio	− 0.150	0.445	− **0.419**	**0.046**	− 0.159	0.502
PaCO_2_	**0.404**	**0.018**	0.257	0.178	0.384	0.053
AST (U/L)	0.302	0.111	**0.456**	**0.013**	0.374	0.050
ALT (U/L)	0.059	0.766	0.092	0.635	0.108	0.586
ALP (U/L)	**0.614**	**< 0.001**	**0.648**	**< 0.001**	**0.458**	**0.014**
G-GT (U/L)	0.200	0.290	0.240	0.219	0.239	0.220
Total bilirubin (mg/dL)	0.312	0.087	0.344	0.073	0.157	0.424
Direct bilirubin (mg/dL)	0.426	0.061	0.228	0.450	− 0.065	0.811
Albumin (g/L)	− **0.610**	**< 0.001**	− **0.456**	**0.013**	− **0.637**	**< 0.001**
LDH (U/L)	**0.725**	**0.007**	**0.812**	**< 0.001**	0.517	0.162
S-CRP (mg/L)	0.196	0.266	**0.540**	**0.001**	**0.403**	**0.030**
S-TNF-α (pg/mL)	− 0.001	0.995	0.261	0.143	**0.410**	**0.025**
S-IL1-β (pg/mL)	− 0.013	0.940	0.025	0.890	− 0.294	0.114
S-IL-6 (pg/mL)	0.217	0.217	**0.386**	**0.027**	0.290	0.120
Leukocytes (× 10^9^/L)	0.343	0.051	**0.379**	**0.033**	− 0.035	0.860
Neutrophils (× 10^9^/L)	0.262	0.148	**0.551**	**0.001**	− 0.110	0.585
Eosinophils (× 10^9^/L)	0.317	0.077	**0.387**	**0.032**	0.203	0.309
Monocytes (× 10^9^/L)	0.221	0.224	− 0.121	0.517	− 0.351	0.072
Lymphocytes (× 10^9^/L)	0.240	0.186	0.004	0.981	0.020	0.921
NLR	− 0.041	0.823	0.307	0.093	− 0.118	0.558
NMR	− 0.071	0.696	**0.502**	**0.004**	0.179	0.370
ICU length of stay	**0.405**	**0.017**	**0.352**	**0.045**	**0.556**	**0.001**
Total hospital length of stay (days)	**0.360**	**0.037**	0.288	0.104	**0.445**	**0.014**

*ALP* alkaline phosphatase, *ALT* alanine transaminase, *APACHE II* acute physiology and chronic health evaluation II, *AST* aspartate aminotransferase, *FiO*_*2*_ fraction of inspired oxygen, *ALP* alkaline phosphatase, *G-GT* gamma-glutamyl transferase, *ICU* intensive care unit, *LDH* lactate dehydrogenase, *NLR* neutrophil–lymphocyte ratio, *NMR* neutrophil–monocyte ratio, *PaCO*_*2*_ partial pressure of arterial carbon dioxide, *PaO*_*2*_ partial pressure of arterial oxygen, *SAPS II* simplified acute physiology score II, *S-CRP* serum C-reactive protein. Significant correlations are highlighted in bold

### U-CysLT during the first week of hospitalization in survivors and non-survivors at 30 days

**Fig. 4:**
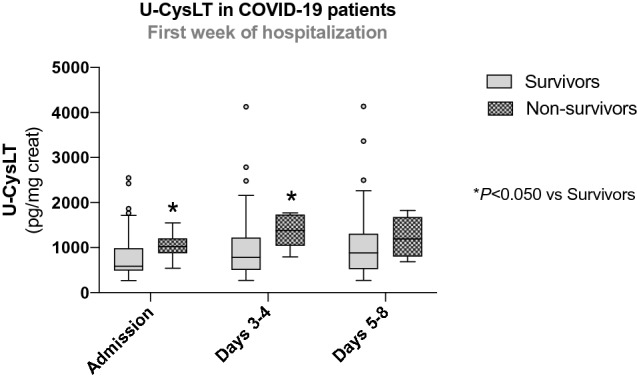
U-CysLT at admission, days 3–4 and 5–8 in COVID-19 survivors and non-survivors (mortality at 30 days). Results are presented in Box-and-Whiskers plot, survivors: admission (*n* = 52), days 3–4 (*n* = 47), days 5–8 (*n* = 40); non-survivors: admission (*n* = 8), days 3–4 (*n* = 6), days 5–8 (*n* = 5). *U-CysLT* urinary cysteinyl leukotrienes, *VV-ECMO* veno-venous extracorporeal membrane oxygenation

Non-survivor COVID-19 patients had significantly higher values of U-CysLT at admission (*p* < 0.010) and at days 3–4 (*p* < 0.010) compared to survivors (Fig. 4). On days 5–8, although non-survivors also presented higher values than survivors, this difference did not reach statistical significance (*p* = 0.148) (Fig. 4).

### Repeated measures multivariate analysis

When considering all COVID-19 patients, we observed a significant positive association between U-CysLT and the most severe clinical presentation, i.e., the critical COVID-19 on VV-ECMO group (*P* = 0.001) (Table 4). We did not find any significant association of U-CysLT with comorbidities (data not shown).

**Table 4:** Repeated measures multivariate models for U-CysLT in COVID-19 patients

U-CysLT (pg/mg creatinine)	Adjusted *β*	95% CI	*p* value
Multivariate analysis—all COVID-19 patients			
Model 1: COVID-19 Group			
Severe	Ref		
Critical	162.45	− 155.54–480.43	0.317
** Critical on VV-ECMO**	**716.55**	**294.49–1138.61**	**0.001**
Multivariate analysis—all critically ill COVID-19 patients^a^			
Model 2: hypertension			
Yes	Ref		
No	67.34	− 401.41–536.10	0.778
Model 3: prior treatment with RAAS inhibitors			
Yes	Ref		
No	415.06	− 90.16–920.29	0.107
Model 4: eGFR (ml/min/1.73 m^2^)	10.76	− 0.140–21.66	0.053
Model 5:** ICU length of stay (days)**	**9.82**	**4.72–14.92**	**< 0.001**
Model 6: **Total hospital length of stay (days)**	**5.47**	**1.15–9.80**	**0.013**
Model 7: **Mortality at 30 days**			
Yes	Ref		
**No**	**− 350.55**	**− 677.11–23.984**	**0.035**

(Adjusted *β*), 95% confidence intervals (95% CI) and *p* value estimated by repeated measures multivariate models with U-CysLT as the dependent variable and adjusted for age and gender. Significant associations are highlighted in bold

*eGFR* estimated glomerular filtration rate, *ICU* intensive care unit, *RAAS* renin–angiotensin–aldosterone system, *Ref* reference, *U-CysLT* urinary cysteinyl leukotrienes, *VV-ECMO* veno-venous extracorporeal membrane oxygenation

^a^Critical + critical on VV-ECMO

Among all critical COVID-19 patient groups, higher U-CysLT values were significantly associated with higher ICU and total hospital length of stay (*p* < 0.001 and *p* = 0.013, respectively) (Table 4). Furthermore, survival at 30 days was associated with significantly lower values of U-CysLT (*p* = 0.035) (Table 4).

### Performance of U-CysLT as a predictor of mortality at 30 days

**Fig. 5:**
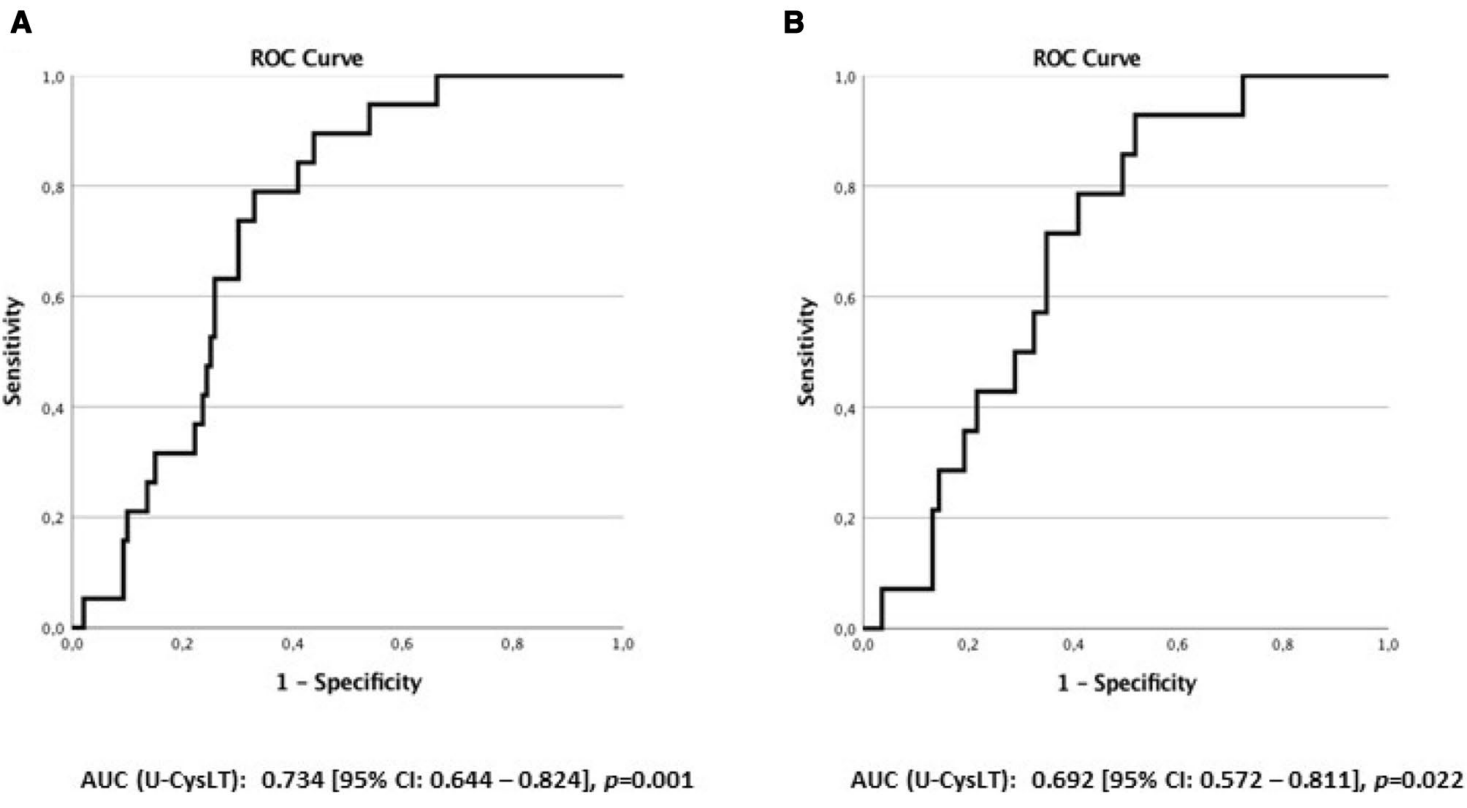
Receiver-operating characteristic (ROC) curves of U-CysLT for discriminating mortality at 30 days: **A** in all COVID-19 patients (*n* = 60); **B** in critically ill patient groups (*n* = 34). *AUC* area under curve, *U-CysLT* urinary cysteinyl leukotrienes, *VV-ECMO* veno-venous extracorporeal membrane oxygenation

We found that U-CysLT had a good ability of discriminating mortality at 30 days among all COVID-patients (AUC (U-CysLT): 0.734 [95% CI: 0.644–0.824], *p* = 0.001) (Fig. 5A) or in critical COVID-19 patient groups (AUC (U-CysLT): 0.692 [95% CI: 0.572–0.811], *p* = 0.022) (Fig. 5B).

## Discussion

Our major findings were that U-CysLT significantly increased throughout hospitalization in both groups of critical COVID-19 patients, with the highest values being observed in those on VV-ECMO support. Furthermore, in VV-ECMO patients, arterial hypertension was also associated with significantly higher U-CysLT values at the end of the first week of hospitalization. Importantly, correlation and repeated measures multivariate analysis showed that, among critical patients, U-CysLT values during the first week of hospitalization were positively associated with prognostic parameters such as ICU length of stay, total hospital length of stay and mortality. This relationship with mortality at 30 days was also evidenced by the markedly higher U-CysLT values during the first hospitalization week in non-survivors when compared to survivors and corroborated by ROC curve analysis that confirmed a good ability of U-CysLT to predict 30-day mortality in COVID-19 patients.

As other causes of ARDS, COVID-19 pneumonia requiring VV-ECMO has a high mortality rate [32–35] but these patients are still scarcely studied. In our study, VV-ECMO patients showed the highest values of U-CysLT on the first week of hospitalization. Although aging has been linked to an imbalance in the production of arachidonic acid metabolites, with a shift favoring CysLT proinflammatory actions [36], it cannot explain our results since patients on VV-ECMO were the youngest COVID-19 patients, which is in accordance to guidelines and similar to that observed in other series of COVID-19 patients on VV-ECMO support [37, 38]. In fact, the younger age of our VV-ECMO patients might have been a major determinant for the low mortality observed in this group. In contrast, the significantly older age of patients with severe COVID-19 might explain the similar mortality found between severe and critical COVID-19 patients, since age appears to be the most relevant risk factor for critical disease and death, with a significantly increased risk at each life decade [39]. In VV-ECMO patients, severe hypoxia and obesity predominance could be partially responsible for the activation of the LT pathway, as already described for in vitro immune and endothelial cells exposed to intermittent hypoxia [40, 41] and in the severest stages of obstructive sleep apnea (OSA) [42, 43]. Body mass index (BMI) appears to be an independent predictor of urinary LTE_4_ values in obese (BMI ≥ 30 kg/m^2^) or overweight (25 < BMI < 30 kg/m^2^) OSA patients, even after adjustment for OSA severity [44]. Furthermore, adipocytes exhibit the ability to synthetize CysLT [45, 46]. However, when comparing U-CysLT in obese and non-obese patients within COVID-19 patient groups, obesity was not associated with higher values of these mediators and in critical COVID-19 patients, it was even associated with lower U-CysLT.

Arterial hypertension has been established as a major risk factor for disease severity and mortality associated with COVID-19, while being also a predictor of poor outcomes in COVID-19 patients [23–25], probably by dysregulation of the renin–angiotensin–aldosterone system (RAAS) and the association with inflammation and immune responses [47]. In our cohort, similarly to other series of COVID-19 critical patients [48], we found that hypertension was the most prevalent comorbidity among severe and critical COVID-19 groups and the second most frequent comorbidity in critical COVID-19 on VV-ECMO group. Noteworthy, our results showed that among critical on VV-ECMO patients, those who were hypertensive had higher U-CysLT values at the end of the first week of hospitalization. LTs are known to play a variety of roles in the cardiovascular system. Specifically, CysLT cause constriction of the smooth muscle, increase capillary permeability and can even decrease cardiac inotropism and coronary blood flow [49, 50]. Recent studies have shown that 5-LOX-derived proinflammatory mediators, including CysLT, may be involved in vascular remodeling contributing to elevated systemic vascular resistance and consequently to higher blood pressure in mice models [50]. Evidence has also shown that selective CysLT_1_ receptor antagonists were able to mitigate angiotensin II-mediated vasoconstriction in several animal and human models [51–53], therefore, suggesting a strong involvement of CysLT in the pathogenesis of angiotensin II-induced hypertension [54]. Given the relationship between CysLT and angiotensin II, along with the fact that treatment with RAAS inhibitors was interrupted during hospitalization in ICU patients, we could hypothesize that the maintenance, whenever possible, of antihypertensive treatment with RAAS inhibitors might be potentially beneficial for critical COVID-19 patients on VV-ECMO.

Both critical COVID-19 groups, but not severe patients, showed a rising pattern of U-CysLT values throughout hospitalization. This might have been caused by an upregulation of 5-LOX in critical COVID-19, but not in severe patients, as previously suggested by other authors who observed an increase in 5-LOX-derived mediators in the sera of ICU COVID-19 patients at day 4 post-admission [55]. Raised concentrations of 5-LOX-derived leukotrienes, such as LTB_4_ and LTE_4_, have also been evidenced in the bronchoalveolar lavage fluid of intubated COVID-19 patients [56].

In our study, critical COVID-19 groups also exhibited significant positive correlations of U-CysLT values with several systemic inflammatory markers and parameters, namely with the proinflammatory cytokines IL-6 and TNF-α, with CRP and with total leukocytes, neutrophils, eosinophils and NMR ratio. Interestingly, this relationship of U-CysLT with the inflammatory status was far more pronounced in critical COVID-19 patients than in septic shock patients recently evaluated by our group [13]. This suggests that U-CysLT could be more implicated in COVID pathogenesis, as also denoted by their association with prognosis. Interestingly, critical patients on VV-ECMO showed a higher number of eosinophils which are a known major source of CysLT [57]. CysLT mediate various eosinophil functions, namely their differentiation, maturation and survival, as well as chemotaxis and protein secretion, with most of the abovementioned functions being mediated by CysLT_1_ receptor [58]. Accordingly, CysLT_1_ receptor antagonists are able to reduce eosinophil recruitment seen in allergic inflammation of the airways [8, 59]. Both eosinopenia and eosinophilia seem to be associated with the dysregulated immune response in COVID-19 patients [60]. While eosinopenia has been associated with poor outcomes in initial acute phases of COVID-19 [60], eosinophil-mediated lung inflammation seems to contribute to the development of critical forms of the disease [61]. Overall, our findings seem to establish a connection between the hyperinflammatory state in critical COVID-19 patients and CysLT, especially during disease progression.

Dysregulation and overactivation of the immune system are probable mechanisms for liver injury in COVID-19 patients [62]. High concentrations of AST, ALT, LDH, total bilirubin and decreased albumin concentrations have been observed in severe forms of COVID-19 disease and are associated with hepatic dysfunction [63]. A cholestatic pattern, such as G-GT and ALP elevation, has also been described in COVID-19 patients [64]. Indeed, we noticed this same pattern in our critical COVID-19 on VV-ECMO patients, with raised concentrations of G-GT and direct bilirubin, as well as lower concentration of serum albumin. Noteworthy, in critical COVID-19 patient groups, U-CysLT were also associated with hepatic injury/dysfunction, being positively correlated with AST and ALP and inversely correlated with albumin. Importantly, we also recently evidenced a positive association between U-CysLT and hepatic injury in septic shock patients, thus reinforcing the contribution of these mediators to liver damage [13].

Ninety-four percent of patients in critical groups were under dexamethasone treatment according to hospital protocol and treatment guidelines [28]. Therefore, we could not evaluate the impact of dexamethasone on U-CysLT values in COVID-19 patients. Nonetheless, several studies have described an inability of both topical and systemic corticosteroids to modulate overall secretion of CysLTs by immune cells [65–68]. In addition, critical groups had a higher percent of patients under antibiotic treatment. Co-infection conditioning antibiotic treatment could be a confounder for the hyperinflammatory state and increased CysLT values. In fact, both lipoteichoic acids from Gram-positive and endotoxin from Gram-negative bacteria appear to induce CysLT production [69, 70]. On the other hand, antibiotics are known modulators of the inflammatory response and their immunomodulatory properties have been described in inflammatory respiratory diseases [71]. Further studies should therefore assess the impact of co-infection and antibiotic treatment on CysLT profile in COVID-19 patients.

U-CysLT values did not distinguish clinical severity at admission, appearing to have a more delayed kinetics in COVID-19 pathophysiology as evidenced by their rising pattern along hospitalization in critical patient groups. Importantly, U-CysLT values during the first week of hospitalization were consistently positively associated with relevant prognostic markers such as ICU length of stay, total hospital length of stay and mortality at 30 days. Non-survivors presented significantly higher values of U-CysLT during the first week of hospitalization and ROC curve analysis showed good performance of U-CysLT to predict short-term mortality among all patients and in critical patient groups. Thus, measurements of U-CysLT throughout the first week of hospitalization might be useful for risk stratification and prognostication, although this should be further studied in larger patient populations.

Overall, our findings are in accordance with the strong association between the hyperimmune state and COVID-19 progression and mortality [6], reinforcing CysLT as plausible mediators in this process, and, most relevantly, as potential therapeutic targets, as previously proposed [7, 72].To date, only a few studies examined CysLT involvement in COVID-19 pathophysiology. One study detected high concentrations of LTE_4_ in bronchoalveolar lavage fluid of patients with critical COVID-19 requiring mechanical ventilation [56]. Another study showed exacerbated macrophage production of CysLT three to five months after onset of non-severe COVID-19 [73]. Moreover, the role of proinflammatory eicosanoids in critical COVID-19 was recently highlighted [74]. Importantly, montelukast, an antagonist of CysLT_1_ receptor, was shown to inhibit platelet activation induced by plasma of COVID-19 patients [75]. This study also suggested that montelukast, if administered in the early phase of disease, could mitigate lung damage in COVID-19 patients by targeting inflammation and immune response, in addition to limiting the thrombotic phenomena that are observed in this disease [75]. In fact, two retrospective studies have already demonstrated that asthmatic patients under treatment with montelukast had fewer episodes of COVID-19 [76] or less episodes of clinical deterioration [77]. Just recently, a retrospective controlled cohort study also showed that the combined treatment with dexamethasone and CysLT receptor antagonists provided a survival advantage in COVID-19 patients with low oxygen saturations [78]. Clinical trials are already on place to test montelukast’s effectiveness on COVID-19 treatment, including COSMO [79] and E-SPREZANZA trials [80] and also a Portuguese randomized, controlled, parallel, open-label trial [81].

Major strengths of our study comprise the measurement of U-CysLT throughout hospitalization and the analysis of an extensive panel of biomarkers related to inflammatory and multisystemic manifestations of COVID-19, as well as the assessment of U-CysLT relationship with severity and prognostic parameters in hospitalized patients, especially in ICU and VV-ECMO patients where investigation is very limited. The use of non-invasive samples and a simple methodology to quantify U-CysLT, along with its consistent association with prognostic parameters, reinforce the clinical usefulness of this biomarker. Limitations of the study include its single-center design, which may condition the existence of a selection bias derived from consecutive recruitment, as well as the small number of patients included. In addition, we were not always able to collect blood and urine samples at all time points from all patients due to the high burden of clinical work during COVID-19 pandemic and also due to patient withdrawal of consent associated with the fear and psychological distress in hospitalized COVID-19 patients.

In conclusion, U-CysLT increases during hospitalization in patients with critical COVID-19, particularly in those on VV-ECMO support. Arterial hypertension, but not obesity, seems to contribute to the raised U-CysLT values at the end of the first week of hospitalization in critically ill patients on VV-ECMO. Importantly, among all critically ill patients, U-CysLT values along the first week of hospitalization were positively associated with outcomes such as ICU length of stay and total hospital length of stay. Moreover, a significant relationship between U-CysLT and mortality at 30 days was also confirmed, with U-CysLT values at first week of hospitalization showing good performance to predict short-term mortality among all patients and in critically ill patients. Thus, they may represent important biomarkers for risk stratification and prognostication in COVID-19, as well as therapeutic targets, especially in critically ill patients.

## Supplementary Information

Below is the link to the electronic supplementary material.

Supplementary file1 Supplementary Fig. 1. Flowchart indicating the number of severe COVID-19 patients analysed at each time point and the reasons for missing data and patient drop out from the study. (TIF 130 KB)

Supplementary file2 Supplementary Fig. 2. Flowchart indicating the number of critical COVID-19 patients analysed at each time point and the reasons for missing data and patient drop out from the study. (TIF 120 KB)

Supplementary file3 Supplementary Fig. 3. Flowchart indicating the number of critical COVID-19 on VV-ECMO patients analysed at each time point and the reasons for missing data and patient drop out from the study. (TIF 115 KB)

## Data Availability

The datasets generated during and/or analysed during the current study are available from the corresponding author on reasonable request.
