# Supplementary figures and images for: Evaluation of urinary cysteinyl leukotrienes as biomarkers of severity and putative therapeutic targets in COVID-19 patients
Source: Inflamm Res. 2023 Jan 8;72(3):475–91. doi: 10.1007/s00011-022-01682-z (PMC9826622; doi:10.1007/s00011-022-01682-z)

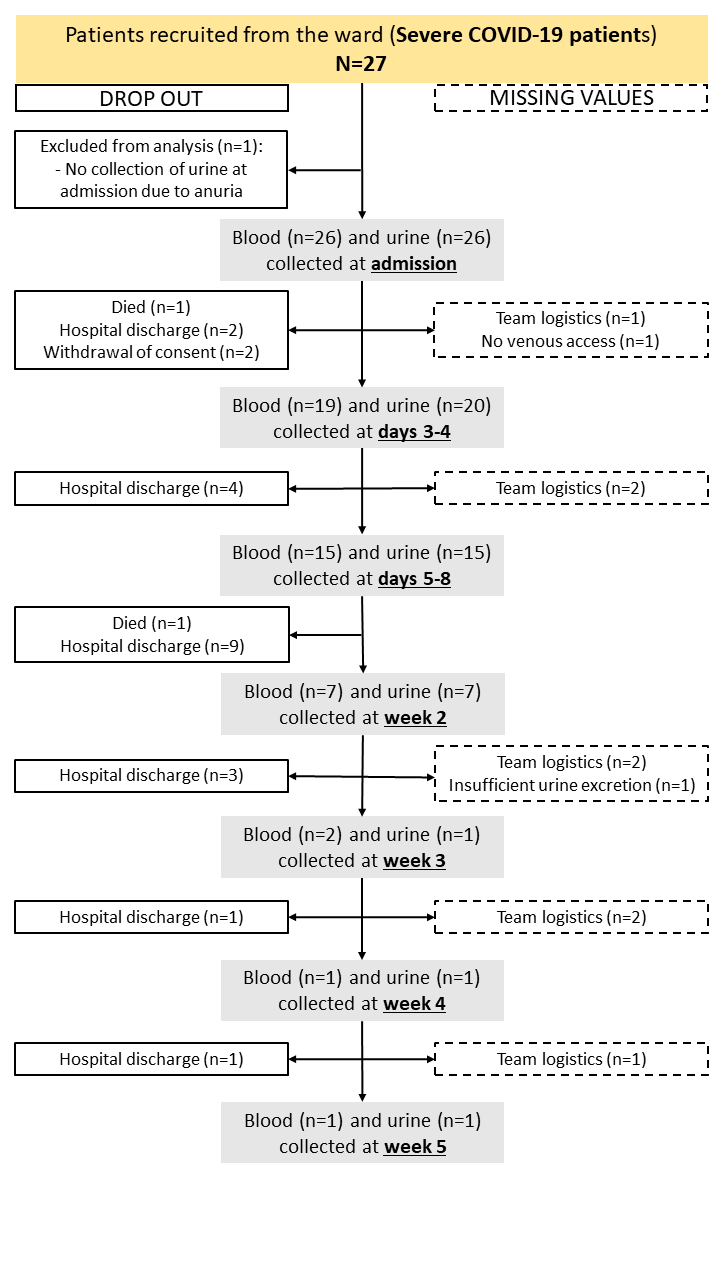

Supplement: Supplementary file 1 — Supplementary file1 Supplementary Fig. 1. Flowchart indicating the number of severe COVID-19 patients analysed at each time point and the reasons for missing data and patient drop out from the study. (TIF 130 KB) [file 11_2022_1682_MOESM1_ESM.tif]

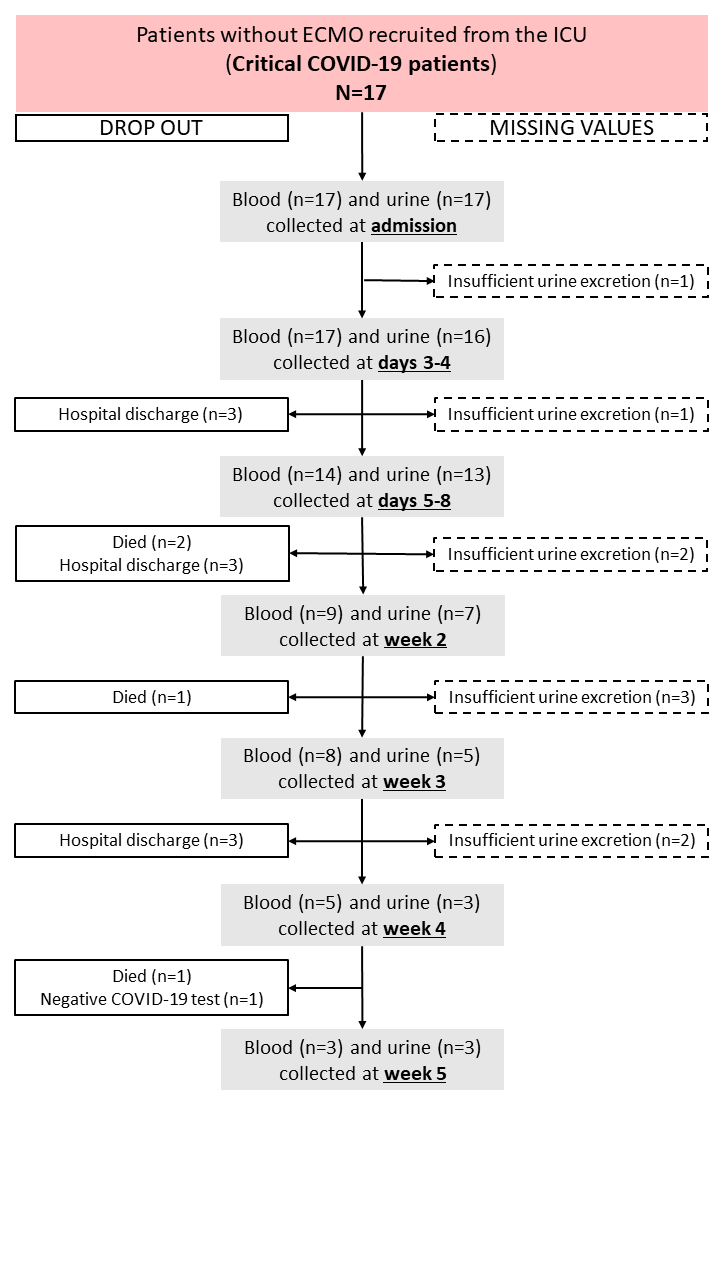

Supplement: Supplementary file 2 — Supplementary file2 Supplementary Fig. 2. Flowchart indicating the number of critical COVID-19 patients analysed at each time point and the reasons for missing data and patient drop out from the study. (TIF 120 KB) [file 11_2022_1682_MOESM2_ESM.tif]

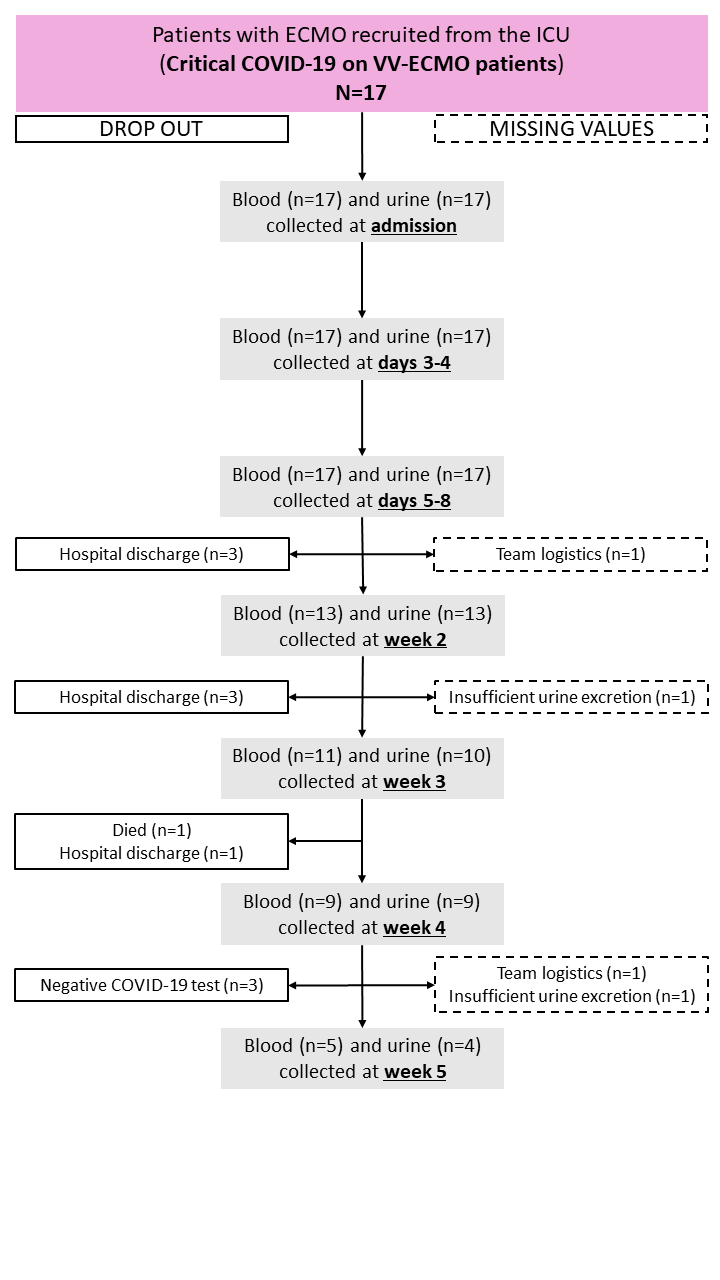

Supplement: Supplementary file 3 — Supplementary file3 Supplementary Fig. 3. Flowchart indicating the number of critical COVID-19 on VV-ECMO patients analysed at each time point and the reasons for missing data and patient drop out from the study. (TIF 115 KB) [file 11_2022_1682_MOESM3_ESM.tif]
